# ﻿Seven new species and four new records of Psychomyiidae (Insecta, Trichoptera) from China

**DOI:** 10.3897/zookeys.1188.112359

**Published:** 2024-01-08

**Authors:** Lang Peng, Zhen Deng, Yu-hua Zhang, Meng Wang, Chang-hai Sun, Bei-xin Wang

**Affiliations:** 1 Department of Entomology, College of Plant Protection, Nanjing Agricultural University, Nanjing 210095, China Nanjing Agricultural University Nanjing China

**Keywords:** Caddisflies, morphology, new geographical records, new synonym, Oriental Region

## Abstract

Seven new species of the family Psychomyiidae Walker, 1852 are described and illustrated from China; they are *Psychomyiashuni***sp. nov.**, *Ps.mangshanensis***sp. nov.**, *Ps.capricornis***sp. nov.**, *Lypesagittalis***sp. nov.**, *Paduniellafasciaria***sp. nov.**, *Pa.sanyaensis***sp. nov.**, and *Tinodesaviformis***sp. nov.** The genus *Lype* is reported for the first time from mainland China. In addition, four psychomyiids are found to be new to the Chinese caddis fauna: *Psychomyiaindra* Malicky & Chantaramongkol, 1993; *Paduniellaandamanensis* Malicky, 1979; *Pa.dendrobia* Malicky & Chantaramongkol, 1993; and *Tinodesgapbona* Johanson & Oláh, 2008. Moreover, *Psychomyiapolyacantha* Li, Qiu & Morse, 2021 is reviewed and synonymized with *Psychomyiaimamiah* Malicky, 2020.

## ﻿Introduction

The family Psychomyiidae Walker, 1852 currently includes 648 extant species in eight genera, of which 202 species are included in the genus *Psychomyia* Latreille, 1829 (Latreille 1829–1830; Laudee et al. 2020; Malicky 2020; Peng et al. 2020; Qiu and Morse 2021; Morse 2023), 316 species in *Tinodes* Curtis, 1834 (Curtis 1834; Malicky 2020; Peng et al. 2022; Morse 2023), 79 species in *Paduniella* Ulmer, 1913 (Ulmer 1913; Malicky et al. 2019; Morse 2023), 23 species in *Eoneureclipsis* (Kimmins 1955; Malicky 2020; Suwannarat et al. 2020; Morse 2023; Peng et al. 2023), 15 species in *Lype* McLachlan, 1878 (McLachlan 1878; Morse 2023), 11 species in *Metalype* Klapálek, 1898 (Klapálek 1898; Morse 2023), and one species each in the genera *Padangpsyche* Malicky, 1993 and *Trawaspsyche* Malicky, 2004 (Malicky 1993, 2004). They are widely distributed in the world’s major biogeographic regions except for the Neotropical and Antarctic regions, of which more than 400 species are in the Oriental Region.

In this study, we describe three new species of the genus *Psychomyia* (*Psychomyiashuni* sp. nov., *Ps.mangshanensis* sp. nov., and *Ps.capricornis* sp. nov.), two of *Paduniella* (*Paduniellafasciaria* sp. nov. and *Pa.sanyaensis* sp. nov.), and one each in *Lype* (*Lypesagittalis* sp. nov.) and *Tinodes* (*Tinodesaviformis* sp. nov). The genus *Lype* is newly reported from mainland China. In addition, the present study provides four new geographical records for China (*Psychomyiaindra* Malicky & Chantaramongkol, 1993; *Paduniellaandamanensis* Malicky, 1979; *Pa.dendrobia* Malicky & Chantaramongkol, 1993; *Tinodesgapbona* Johanson & Oláh, 2008) and suggests a species synonymy (*Psychomyiapolyacantha* Li, Qiu & Morse, 2021 (in Qiu and Morse 2021), syn. nov. of *Psychomyiaimamiah* Malicky, 2020).

## ﻿Materials and methods

### ﻿Sample collection

Adult specimens were captured in 100% ethanol by light traps with ultraviolet light bulbs and by Malaise trap. All specimens were stored in 95% ethanol immediately after being sorted into species. All specimens and collectors or collecting institutions are listed in Table 1; the collectors including Dr Christy Jo Geraci (CJG), Mr Wei Cao (CW), Ms Xiao Chen (CX), Dr Xin-yu Ge (GXY), Prof. John C. Morse (JCM), Mr Kun Jiang (JK), Mr Wei Han (HW), Dr You-wen Li (LYW), Ms Lang Peng (PL), Dr Hai-tian Song (SHT), Prof. Lian-fang Yang (YLF), Mr Hao-ming Zang (ZHM), Ms Jin Zhu (ZJ), Prof. Xin Zhou (ZX), Institute of Zoology, Guangdong Academy of Sciences (**GDAS**).

**Table 1.** Detailed information on Psychomyiidae specimens collected in China. Specific dates are for specimens collected by light trap, while a date range represents specimens collected by Malaise trap over several days.

No.	Province/ municipality	City/ county	Site	Geographic coordinates	Elevation (m)	Date	Collector(s)	Specimens
1	Fujian	Longyan	Liangyeshan National Nature Reserve	25°12.37'N, 117°11.03'E	750	2-vii-2021	ZJ	*Ps.capricornis* 2♂
	福建	龙岩	梁野山国家级自然保护区				HW	
2	Gansu	Wenxian	Bifeng Gully	32°44.72'N, 105°14.64'E*	650	16-vi-1998	YLF	*Ps.imamiah* 6♂
	甘肃	文县	碧峰沟					
3	Guangdong	Huizhou	Yuguishan Nature Reserve	22°25.80'N, 113°26.39'E	290	17-ix–22-x-2020	GDAS	*T.gapbona* 2♂
	广东	惠州	玉桂山自然保护区					
4	Guangdong	Zhaoqing	Dinghushan National Nature Reserve	23°09.50'N, 112°32.46'E	170	9-ix–9-x-2021	GDAS	*T.gapbona* 20♂
	广东	肇庆	鼎湖山国家级自然保护区					
5	Hainan	Ledong	Jianfengling National Forest Park, Rainforest Valley	18°44.72'N, 108°56.08'E	640	17-iv-2019	SHT	*Ps.indra* 1♂
	海南	乐东	尖峰岭国家森林公园，雨林谷					
6	Hainan	Sanya	Tangta reservoir	18°24.55'N, 109°23.27'E	240	24-vii-2022	PL	*Pa.fasciaria* 26♂16♀
	海南	三亚	汤他水库				ZHM	
7	Hainan	Sanya	Hongxinxi River	18°27.78'N, 109°27.88'E	150	22-vii-2022	PL	*Pa.sanyaensis* 3♂
	海南	三亚	红新溪河				ZHM	
8	Hainan	Sanya	Fuwan reservoir	18°16.80'N, 109°28.94'E	60	25-vii-2022	PL	*Pa.sanyaensis* 2♂
	海南	三亚	福万水库				ZHM	*T.aviformis* 1♂
9	Hunan	Chenzhou	Mangshan National Forest Park	24°58.80'N, 112°55.65'E	730	1-ix-2020	CW	*Ps.mangshanensis* 11♂
	湖南	郴州	莽山国家森林公园					
10	Hunan	Shaoyang	Shunhuangshan National Forest Park	26°23.78'N, 111°00.47'E	750	22-viii-2020	CW	*Ps.shuni* 2♂
	湖南	邵阳	舜皇山国家森林公园					
11	Hunan	Shaoyang	Yaorenping hydropower station	26°14.95'N, 110°30.26'E	900	24-v-2021	PL	*Ps.shuni* 1♂
	湖南	邵阳	瑶人坪水电站					
12	Hunan	Shaoyang	Jiuxi Bamboo Tower Villa	26°24.39'N, 110°05.68'E	630	25-v-2021	PL	*L.sagittalis* 2♂
	湖南	邵阳	九溪竹楼山庄					
13	Hunan	Shaoyang	Guanyinxing	26°24.77'N, 110°05.39'E	550	28-v-2021	PL	*L.sagittalis 1*♂
	湖南	邵阳	观音形					
14	Sichuan	Kangding	Dadu River, Wasigou	30°04.53'N, 102°09.61'E	1430	29-vi-2005	ZX	*Ps.imamiah* 16♂
	四川	康定	大渡河，瓦斯沟				CJG	
15	Sichuan	Pingwu	tributary of Fujiang, 19 km E of Pingwu downtown	32°24.72'N, 104°45.49'E	1090	27-vi-1990	JCM	*Ps.imamiah* 200+♂
	四川	平武	涪江支流，平武县东19千米					
16	Sichuan	Pingwu	17 km E of Ping-wu trib. of Fujianghe	32°24.48'N, 104°44.36'E*	1090	27-vi-1990	YLF	*Ps.imamiah* 200+♂
	四川	平武	涪江河支流，平武县东17千米				LYW	
17	Sichuan	Yibin	Xining River	28°41.15'N, 103°45.97'E	370	12-v-2020	GXY	*Pa.dendrobia* 1♂
	四川	宜宾	西宁河				CX	
18	Yunnan	Jinghong	Yunjinghong Street G214	22°01.75'N, 100°52.12'E	660	26-vii-2021	JK	*Pa.andamanensis* 1♂
	云南	景洪	G214国道，允景洪街道					

* indicates the lack of original records for geographic coordinates; the data are based on location information.

### ﻿Morphological study

Male abdomens used for illustrations were cleared with 10% NaOH solution and heated to 90 °C for 10 min to remove all the non-chitinous tissues. Then the cleaned genitalia were rinsed in distilled water and mounted on a depression slide with lactic acid for examination. Genitalia structures of males were traced with the pencil using a Nikon Eclipse 80i microscope equipped with a camera lucida. Pencil drawings were scanned with an Epson Perfection (V30 SE) scanner, then placed as templates in Adobe Photoshop v. 19.0 (Adobe Inc 2018) software and inked digitally with a Wacom CTL-671 tablet to produce final illustrations. Then each abdomen was stored in a microvial together with the remainder of the specimen in 95% ethanol. All specimens are deposited in the Insect Collection, Nanjing Agricultural University, Nanjing, Jiangsu Province, P.R. China (NJAU).

### ﻿Terminology

The terminology of the male genitalia for the genus *Psychomyia* mainly follows Schmid (1998), with the adoption of the term “mesal ridge” from Qiu and Morse (2021) to refer to the piece along the inner side of each superior appendage; the term “basoventral process” refers to a single or paired protruding structure at the base of the phallotheca, and “basal process” refers to the paired protruding structure at the base of the superior appendages. Terminology for the genus *Lype* largely follows Schmid (1998), with the adoption of the term “subapical projection of aedeagus” from Arefina (2002) to refer to the dorsal process near the distal end of the aedeagus. Terminology for the genus *Paduniella* follows Li and Morse (1997), and that for the genus *Tinodes* follows Peng et al. (2022). To ensure coherency, “superior appendages” are used to refer to the “preanal appendages”, and “coxopodite” and “harpago” are used to refer to the first and second segments of the inferior appendages, respectively.

## ﻿Results

### ﻿Taxonomy

#### ﻿ Psychomyia shuni

Taxon classificationAnimaliaTrichopteraPsychomyiidae

Peng & Sun sp. nov.

BE853E77-FD9D-542B-A73B-FFB525BD6D5C

https://zoobank.org/1FC53A2C-FA77-40B1-A8BC-124A3B949D5E

Fig. 1A–D

##### Type materials.

***Holotype***: China • 1♂; Hunan Province, Shaoyang City, Xinning County, Shunhuangshan National Forest Park; 26°23.78'N, 111°00.47'E; alt. 750 m; 22-viii-2020; light trap; W. Cao leg.; NJAU Tricho-20200822-0001. ***Paratypes***: China • 1♂; same data as holotype; NJAU Tricho-20200822-0002 • 1♂; Hunan Province, Shaoyang City, Chengbu County, Yaorenping hydropower station; 26°14.95'N, 110°30.26'E; alt. 900 m; 24-v-2021; light trap; L. Peng leg.; NJAU Tricho-20210524-0001.

##### Diagnosis.

This species is unique among *Psychomyia* in that the coxopodites and the harpagones are completely fused and together form an S or Z shape in ventral view.

##### Description.

**Figure 1.**
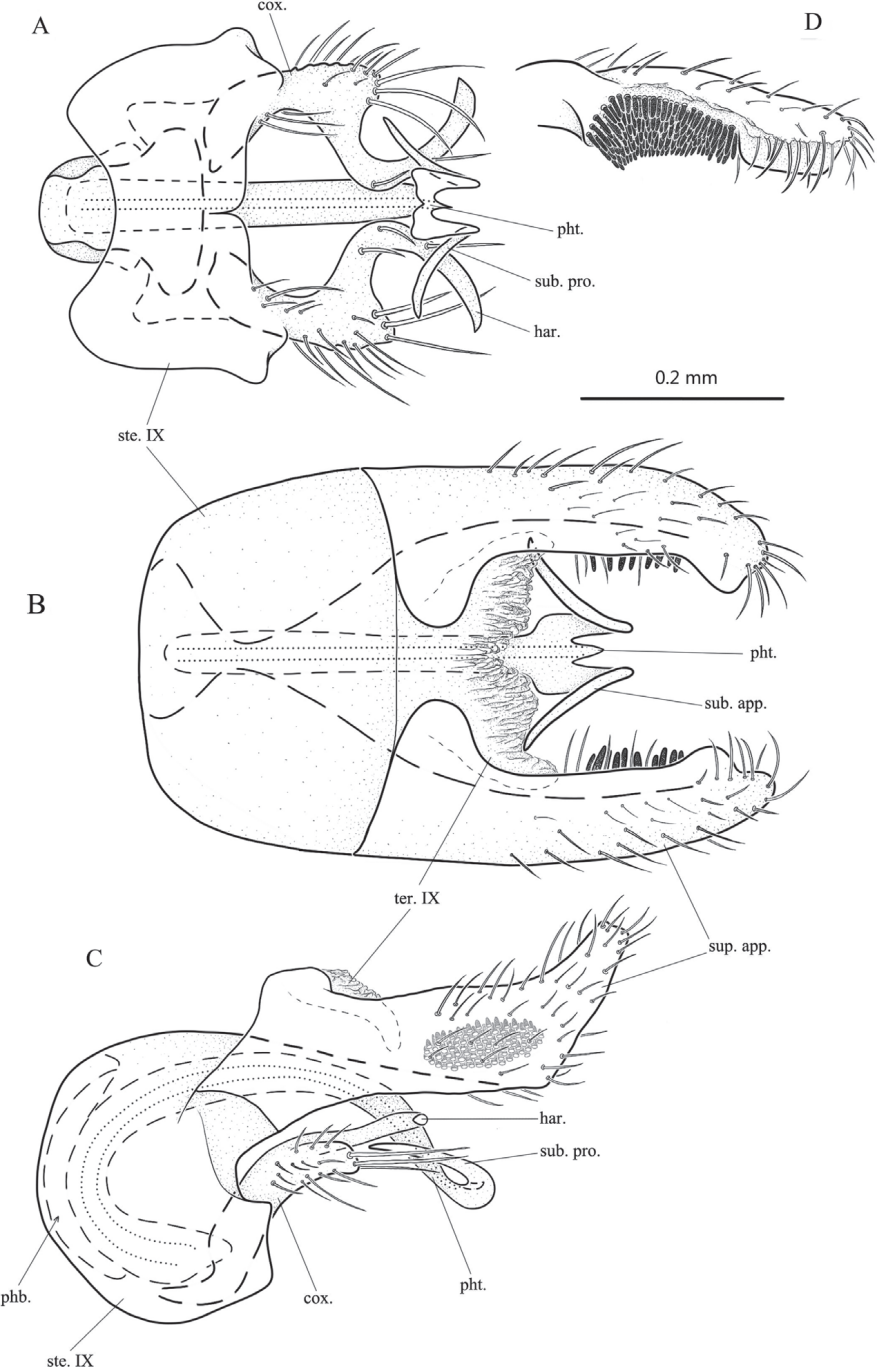
*Psychomyiashuni* sp. nov., male adult, holotype **A** genitalia, ventral **B** genitalia, dorsal **C** genitalia, left lateral **D** superior appendage, ventral. Abbreviations: ste. IX = sternum IX; ter. IX = tergum IX; sup. app. = superior appendage (paired); cox. = coxopodite (paired); har. = harpago (paired); phb. = phallobase; pht. = phallotheca; sub. pro. = subapical process of phallic apparatus.

**Male.** Length of each forewing 3.6–3.8 mm (*n* = 3); holotype 3.7 mm. Specimens in alcohol with compound eyes black; body brown dorsally and light yellow ventrally. Forewings with forks II–V present, hind wings with forks II, III, and V present. ***Genitalia*.** Sternum IX nearly trapezoidal with anterior margin concave in ventral view (Fig. 1A); rounded, semicircular in lateral view (Fig. 1C). Tergite IX produced from dorsoposterior margin of sternum IX, membranous, somewhat bowl-shaped with apical margin sinuate and partially hidden under two superior appendages in dorsal view (Fig. 1B), tongue-shaped in lateral view (Fig. 1C). Superior appendages in dorsal view wide and long, each with larger mesobasal lobe and smaller subapical lobe both directed mesad and with apex truncate (Fig. 1B), twisted and clavate, each with inner side having cluster of spines subapically in ventral view (Fig. 1D); subrectangular in lateral view, each with blunt basodorsal process, angled posterodorsad at 2/3 length to truncate apex (Fig. 1C). Phallobase slender, basally directed anterad and evenly recurved posterad in lateral view (Fig. 1C). Phallotheca tubular, basally directed anterad, evenly recurved dorsad, caudad at midlength, and posteroventrad distally; apically depressed and trifurcate, acute mesally and with pair of long, subapical processes projecting caudad, then recurved dorsad and diverging anterolaterad (Fig. 1A–C). Inferior appendages in ventral view sigmoid (Fig. 1A), in lateral view strongly C-shaped (Fig. 1C); coxopodites elongate-rectangular in lateral view, heavily setose, and fused with slender harpago (Fig. 1C).

##### Etymology.

Latin noun in genitive singular. The new species is named after Shun, a leader of tribal alliances in ancient China, who is considered an important founder of the Chinese civilization. Moreover, the holotype and one of the paratypes were collected at Mt Shun-huang, the mountain named after Shun.

##### Distribution.

China (Hunan).

#### ﻿ Psychomyia mangshanensis

Taxon classificationAnimaliaTrichopteraPsychomyiidae

Peng & Sun sp. nov.

D58141AD-FDEE-5749-93D9-5C39AB2B91D4

https://zoobank.org/2D8150C9-717B-46BF-A82D-E3DFDB7EA29A

Fig. 2A–C

##### Type materials.

***Holotype***: China • 1♂; Hunan Province, Chenzhou City, Yizhang County, Mangshan National Forest Park; 24°58.80'N, 112°55.65'E; alt. 730 m; 1-ix-2020; light trap; W. Cao leg.; NJAU Tricho-20200901-0001. ***Paratypes***: China • 10♂; same data as holotype; NJAU Tricho-20200901-0002 to Tricho-20200901-00011.

##### Diagnosis.

This species is similar to *Psychomyiacuspidata* Li, Qiu & Morse, 2021 from China (Qiu and Morse 2021). However, *P.mangshanensis* sp. nov. can be easily distinguished by the following characteristics: (1) each superior appendage has a small triangular protrusion in the middle of the ventral margin in lateral view, which is missing in *P.cuspidata*; (2) the basal process of each superior appendage is unbranched and with thick spine apically, whereas each superior appendage is two-branched, each branch has a thick spine apically in *P.cuspidata*; and (3) the phallotheca is slightly wavy at midlength in lateral view but with an obtuse angle in *P.cuspidata*.

##### Description.

**Figure 2.**
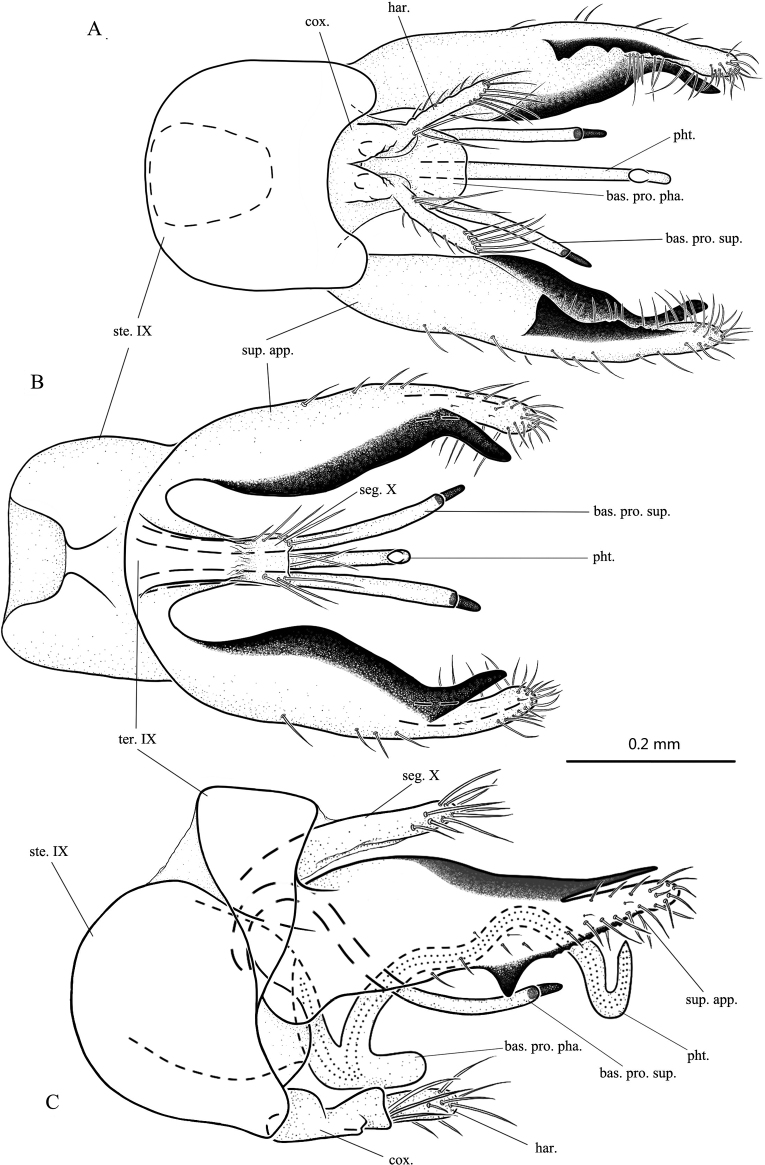
*Psychomyiamangshanensis* sp. nov., male adult, holotype **A** genitalia, ventral **B** genitalia, dorsal **C** genitalia, left lateral. Abbreviations: ste. IX = sternum IX; ter. IX = tergum IX; seg. X = segment X; sup. app. = superior appendage (paired); bas. pro. sup. = basal process of superior appendage (paired); cox. = coxopodite (paired); har. = harpago (paired); pht. = phallotheca; bas. pro. pha. = basoventral process of phallic apparatus.

**Male.** Length of each forewing 2.8–3.1 mm (*n* = 10), holotype forewing 3.0 mm. Specimens in alcohol with compound eyes black; body dark brown dorsally and light brown ventrally. Forewings each with forks II–V present, and hind wings each with forks II and V present. ***Genitalia*.** Sternum IX subrectangular in ventral, dorsal, and lateral views (Fig. 2A, C). Tergite IX short and triangular in dorsal and lateral views (Fig. 2B, C). Division between tergite IX and segment X indiscernible in dorsal view (Fig. 2B) but distinguished by membrane in lateral view (Fig. 2C). Segment X parallel-sided, same width as apex of tergite IX, apically truncate in dorsal view, digitate in lateral view (Fig. 2C); with several long thick apical setae in dorsal and lateral views (Fig. 2B, C). Superior appendages well developed in lateral view, each tapering from base towards apex, divided into one narrow dorsomesal branch and one broad ventrolateral branch subapically; dorsomesal branch sclerotized and bare, acute in lateral view, ventrolateral branch setose about twice as wide as upper branch in lateral view (Fig. 2C); in ventral and dorsal views (Fig. 2A, B), dorsomesal branches of superior appendages angled mesad, ventrolateral branches curved slightly mesad; paired basal processes of superior appendages tubular, and slender, each with thick spine apically; in lateral view each with base directed dorsad, then recurved posteroventrad and evenly curved caudad (Fig. 2C); in ventral and dorsal views each slightly curving outwards (Fig. 2A, B). Phallobase slender, lanceolate in lateral view (Fig. 2C). Phallotheca tubular, with base produced caudad in lateral view (Fig. 2C), main portion sinuate, with apex hooked dorsad; phallotheca stick-like in ventral view (Fig. 2A), basoventral process plate-shaped, three times wider than main portion of phallotheca. Inferior appendages extending posterolaterad; coxopodites subtriangular, with their bases fused in ventral view (Fig. 2A); subrectangular, about 3 times as long as tall with middle of dorsal margin concave in lateral view (Fig. 2C); harpagones setose, arising from apices of coxopodites, fingerlike (Fig. 2A, C).

##### Etymology.

Latin feminine adjective *mangshanensis*, referring to the type locality.

##### Distribution.

China (Hunan).

#### ﻿ Psychomyia capricornis

Taxon classificationAnimaliaTrichopteraPsychomyiidae

Peng & Sun sp. nov.

E10F0B8B-0045-5FB5-9BF2-BC1082145BDD

https://zoobank.org/8B56550A-37E4-45D0-832F-BC73AB818566

Fig. 3A–D

##### Type materials.

***Holotype***: China • 1♂; Fujian Province, Longyan City, Wuping County, Liangyeshan National Nature Reserve; 25°12.37'N, 117°11.03'E; alt. 750 m; 2-vii-2021; light trap; J. Zhu & W. Han leg.; NJAU Tricho-20210702-0001. ***Paratype***: China • 1♂; same data as holotype; NJAU Tricho-20210702-0002.

##### Diagnosis.

This species is similar to *Psychomyiashuni* sp. nov. from China in having the superior appendages with dense spines mesally and in having well-developed subapical processes on the phallic apparatus. However, *P.capricornis* sp. nov. can be easily distinguished by the long, slender processes arising from the bases of the coxopodites, which are absent in *P.shuni*.

##### Description.

**Figure 3.**
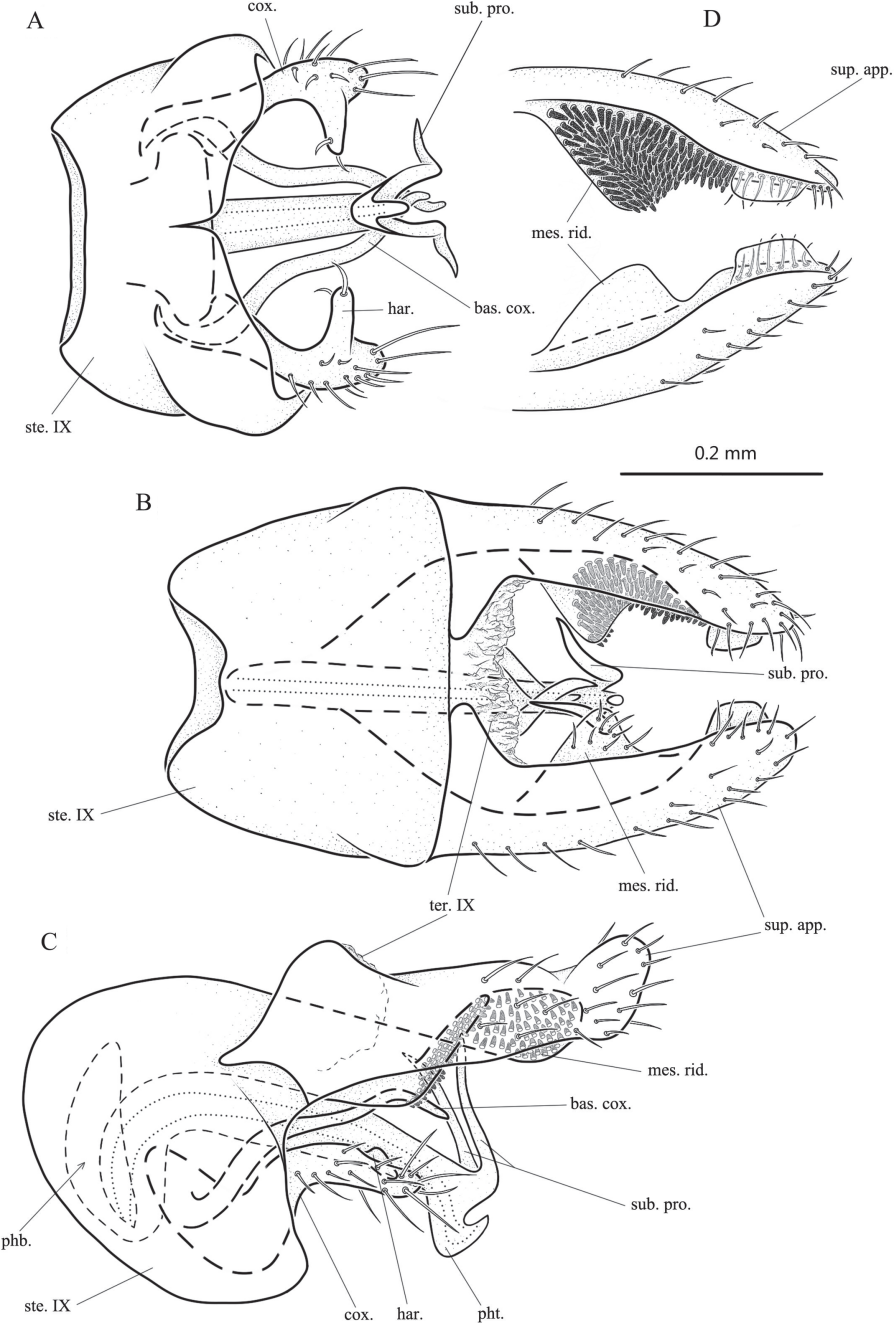
*Psychomyiacapricornis* sp. nov., male adult, holotype **A** genitalia, ventral **B** genitalia, dorsal **C** genitalia, left lateral **D** superior appendages, ventral (mesal spines omitted on right superior appendage). Abbreviations: ste. IX = sternum IX; ter. IX = tergum IX; sup. app. = superior appendage (paired); mes. rid. = mesal ridge of a superior appendage (paired); cox. = coxopodite (paired); bas. cox. = basal process of coxopodite (paired); har. = harpago (paired); phb. = phallobase; pht. = phallotheca; sub. pro. = subapical process of phallic apparatus.

**Male.** Length of each forewing 3.5–3.7 mm (*n* = 2), holotype forewing 3.7 mm. Specimens in alcohol with compound eyes black, body brown dorsally, light yellow ventrally. Forewings with forks II–V present, hind wings with forks II, III, and V present. ***Genitalia*.** Sternum IX nearly trapezoidal with anterior margin concave in dorsal view (Fig. 3B); rounded anteriorly, subelliptical in lateral view (Fig. 3C); with posterior margin narrowly notched mesally in ventral view (Fig. 3A). Tergite IX membranous with irregular posterior margin in dorsal view (Fig. 3B); hemispherical in lateral view (Fig. 3C). Superior appendages elongate-triangular in dorsal view (Fig. 3B); subrectangular in lateral view (Fig. 3C), anterior margin of each produced into apodeme, distal half setose, upper margin sinuate and lower margin straight, with apex oblique and rounded; each with base produced dorsomesad (Fig. 3B, C); mesal ridge cambered and subtriangular with ventral side covered with spines (Fig. 3B, D); elliptical in lateral view (Fig. 3C); and with subapex produced into blunt process mesally in ventral and dorsal views (Fig. 3B, D). Phallobase hemispherical in lateral view (Fig. 3C). Phallotheca directed dorsad basally, then curved dorsocaudad about 90°, subapically with dorsal margins produced into pair of slender and apically acute subapical processes (or “horns”), and distal end hooked dorsad (Fig. 3C); in ventral and dorsal views subapical processes curved outwards at middle (Fig. 3A). Coxopodites setose, each with subapical harpago produced inwards as triangular process in ventral view (Fig. 3A); inconspicuous in lateral view (Fig. 3C). Long, slender, bare process arising from base of each coxopodite, sinuate, apically acute in ventral and lateral views (Fig. 3A, C); extending far beyond apex of coxopodite and harpago, and with distal ends of opposing processes crossed above phallotheca in ventral and dorsal views (Fig. 3A, B).

##### Etymology.

The Latin feminine adjective *capricornis* means “goat’s horn”, referring to the shape of the pair of subapical processes on the phallic apparatus.

##### Distribution.

China (Fujian).

#### ﻿ Lype sagittalis

Taxon classificationAnimaliaTrichopteraPsychomyiidae

Peng & Sun sp. nov.

DB6F042F-7906-5B69-A33A-DD6D4E3D8D8B

https://zoobank.org/5375CC60-19B5-4322-B59A-4856D5F5F6F5

Fig. 4A–E

##### Type materials.

***Holotype***: China • 1♂; Hunan Province, Shaoyang City, Suining County, Jiuxi Bamboo Tower Villa; 26°24.39'N, 110°05.68'E; alt. 630 m; 25-v-2021; light trap; L. Peng leg.; NJAU Tricho-20210525-0001. ***Paratypes***: China • 1♂; same data as holotype; NJAU Tricho-20210525-0002 • 1♂; Hunan Province; Shaoyang City, Suining County, Guanyinxing; 26°24.77'N, 110°05.39'E; alt. 550 m; 28-v-2021; light trap; L. Peng leg.; NJAU Tricho-20210528-0001.

##### Diagnosis.

This species is similar to *Lypelubaretsi* Arefina, 2005 from Russia. However, the new species can be easily distinguished by the following characteristics: (1) longitudinally, sternum IX of *L.sagittalis* sp. nov. is subtriangular in lateral view, rather than subrectangular in *L.lubaretsi*; (2) each of the coxopodites of the new species is subcircular in lateral view, but elliptical in *L.lubaretsi*; (3) the fused coxopodites in ventral view have a narrow mesal notch in the new species, rather than with a wide mesal notch in *L.lubaretsi*; (4) the aedeagus is sagittal, with its apex truncate in dorsal view and pipe-shaped in lateral view in *L.sagittalis* sp. nov., but nearly triangular with apex broad in ventral view, triangular with a sharp apex in lateral view in *L.lubaretsi*.

##### Description.

**Figure 4.**
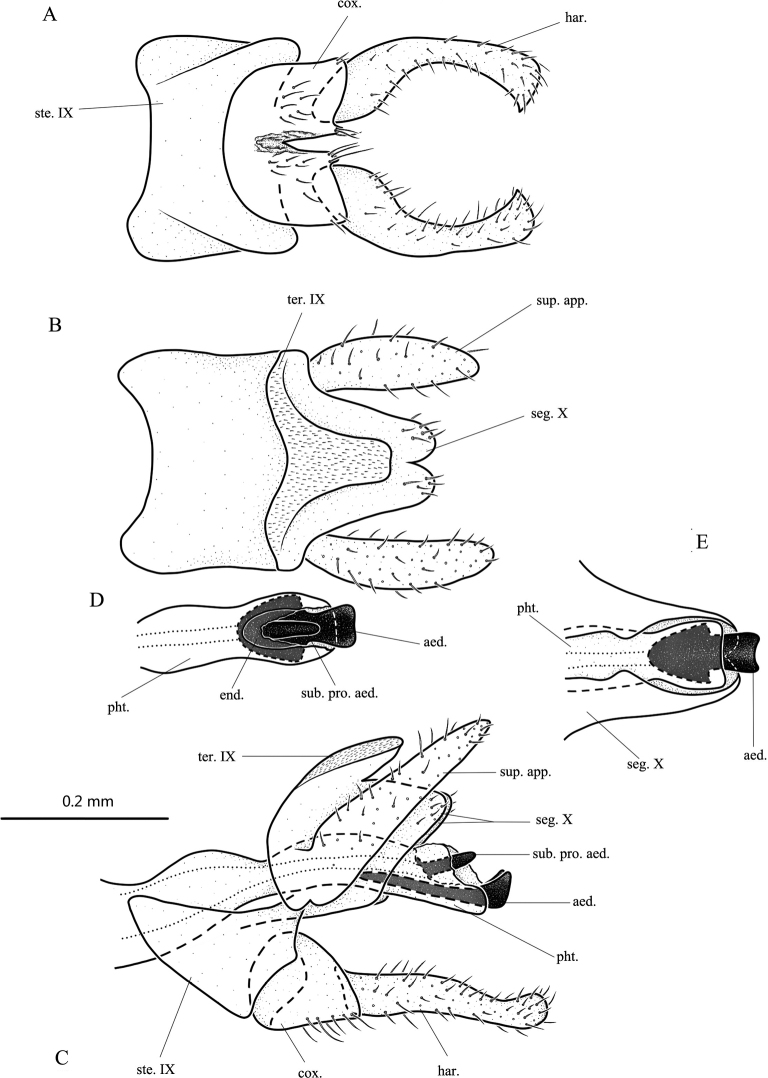
*Lypesagittalis* sp. nov., male adult, holotype **A** genitalia, ventral **B** genitalia, dorsal **C** genitalia, left lateral **D** phallotheca apex, dorsal **E** phallotheca apex and segment X, ventral. Abbreviations: ste. IX = sternum IX; ter. IX = tergum IX; seg. X = segment X; sup. app. = superior appendage (paired); cox. = coxopodite (paired); har. = harpago (paired); pht. = phallotheca; aed. = aedeagus; sub. pro. aed. = subapical projection of aedeagus.

**Male.** Length of each forewing 4.2–4.4 mm (*n* = 3), holotype 4.4 mm. Specimens in alcohol with compound eyes black; antennae, legs, and thorax brown; wings light brown without any distinctive markings; abdomen dark brown dorsally, pale yellow ventrally. ***Genitalia***: Sternum IX subrectangular, anterior margin shallowly concave, posterior margin more deeply concave in ventral view (Fig. 4A); subtriangular in lateral view (Fig. 4C). Tergite IX covered with fine microchaetae, subtriangular with distal end truncate in dorsal view (Fig. 4B); tongue-shaped in lateral view (Fig. 4C), broadly fused with segment X. Segment X subrectangular and almost surrounding phallic apparatus in lateral and ventral views (Fig. 4C, E); apex excised mesally in dorsal view (Fig. 4B). Superior appendages elongate-oval in dorsal view (Fig. 4B); lanceolate, tilted posterodorsad in lateral view (Fig. 4C). Coxopodites each subcircular in lateral view (Fig. 4C); bases fused with each other in ventral view and with narrow notch between them apically about half their length, each with setose posteromesal corner produced caudad (Fig. 4A). Harpagones twice as long as coxopodites, each more-or-less parallel-sided, each with lower margin slightly concave, upper margin sinuate in lateral view (Fig. 4C); the pair divergent basally, evenly curved laterad and mesad to become somewhat forcipiform, with bases and distal ends enlarged in ventral view (Fig. 4A). Phallotheca massive, constricted at middle in lateral view (Fig. 4C), with apical margin membranous. Aedeagus sclerotized, depressed, broad in dorsal and ventral views (Fig. 4D, E); pipe-shaped in lateral view (Fig. 4C). Subapical projection of aedeagus sclerotized, finger-like in lateral and dorsal views (Fig. 4C, D).

##### Etymology.

The Latin feminine adjective *sagittalis*, meaning “arrow-shaped”, and refers to the shape of the aedeagus in dorsal and ventral views.

##### Distribution.

China (Hunan).

#### ﻿ Paduniella fasciaria

Taxon classificationAnimaliaTrichopteraPsychomyiidae

Peng & Sun sp. nov.

6E73EB24-E115-5AAA-8115-B3C56C2B68F5

https://zoobank.org/80C19FBF-FCB6-457E-80BA-E585E4E4F6FE

Fig. 5A–E

##### Type materials.

***Holotype***: China • 1♂; Hainan Province, Sanya City, Tianya district, Tangta reservoir; 18°24.55'N, 109°23.27'E; alt. 240 m; 24-vii-2022; light trap; L. Peng & H. Zang leg.; NJAU Tricho-20220724-0001. ***Paratypes***: 25♂, 16♀; same data as holotype; NJAU Tricho-20220724-0002 to Tricho-20220724-0042.

##### Diagnosis.

This species is similar to *Paduniellasampati* Malicky & Chantaramongkol, 1993 from Thailand in having the superior appendages furcated at their bases in dorsal view and in the shape of the phallic apparatus in the lateral view. However, *P.fasciaria* sp. nov. can be easily distinguished by the possession of a slender median process.

##### Description.

**Figure 5.**
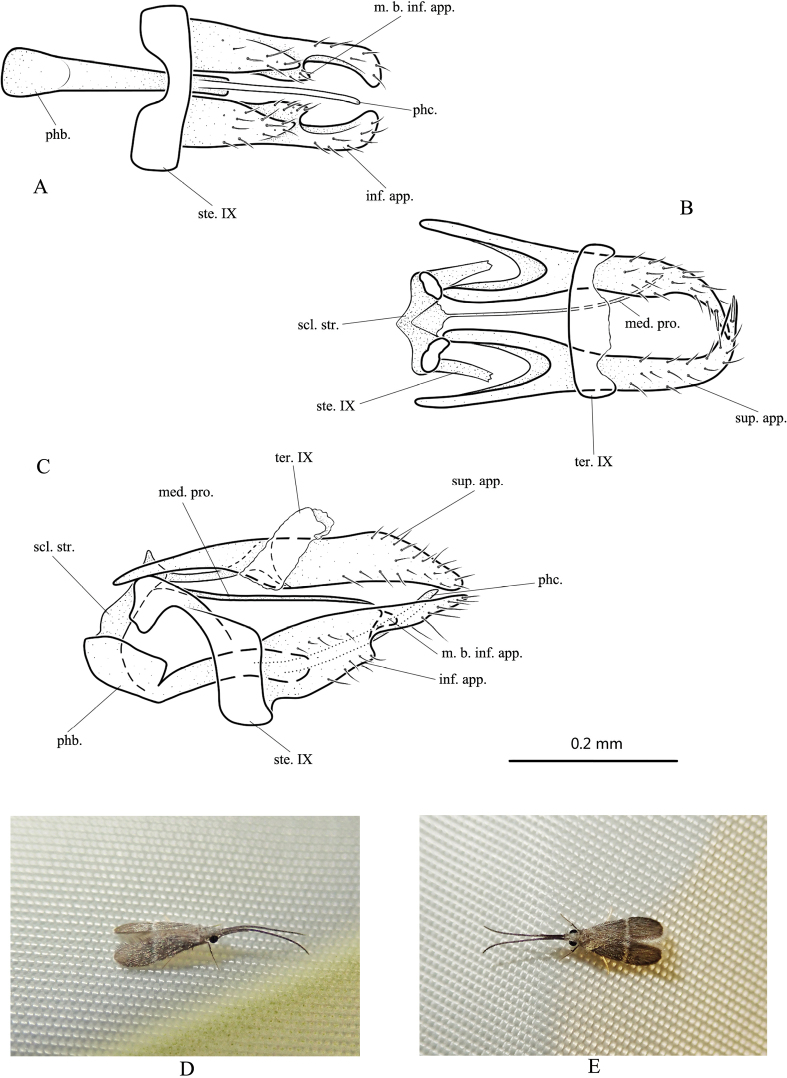
*Paduniellafasciaria* sp. nov., male adult, holotype (**A–C**) and paratypes (**D, E**) **A** genitalia, ventral **B** genitalia, dorsal **C** genitalia, left lateral **D** male adult habitus, right lateral **E** male adult habitus, dorsal. Abbreviations: ste. IX = sternum IX; ter. IX = tergum IX; sup. app. = superior appendage (paired); med. pro. = median process; inf. app. = inferior appendage (paired); m. b. inf. app. = mesal branch of inferior appendage (paired); scl. str. = sclerotized strip; phb. = phallobase; phc. = phallicata. The scale bar refers to **A–C**.

**Male.** Length of each forewing 2.6–3.0 mm (*n* = 10), holotype 2.9 mm. Specimens with compound eyes black, antennae approximately of same length as forewings; body brown; head, bases of antennae, thorax covered with brown, short hair; wings mostly covered with brown, short hair; each forewing with transversal white band at middle (Fig. 5D, E). ***Genitalia*.** Sternum IX subrectangular with anterodorsal angle produced into subrectangular process in lateral view (Fig. 5C); and transversely elongate-rectangular with anterior margin having deep U-shaped incision in ventral view (Fig. 5A). Tergum IX membranous, with base fused with superior appendages, somewhat clavate in lateral view (Fig. 5C), directed posterodorsad; transversely subrectangular in dorsal view, with anterior margin slightly convex and posterior margin undulated (Fig. 5B). Superior appendages large, forming a parallelogram shape in lateral view (Fig. 5C); in dorsal view (Fig. 5B) each with basal portion furcate, basolateral lobe slightly longer than inner one, tapering distally with apex curved mesad and crossing apex of opposing superior appendage, setose subapically. Sclerotized strips slightly clavate in lateral view (Fig. 5C) and somewhat V-shaped in dorsal view (Fig. 5B). Median process mostly slender with sharp apex, insertion between sclerotized strips broad in dorsal view (Fig. 5B). Inferior appendages each with basal half broad, then abruptly narrowed at mid length and tapered towards apex in lateral view (Fig. 5C); basal half broad, then abruptly narrowed, with apex slightly enlarged and curved mesad in ventral view (Fig. 5A), mesal branch lamellar, setose, arising from middle part of inner surface (Fig. 5B, C). Phallobase well developed, basally clavate in lateral view (Fig. 5C); with upper margin having deep incision in ventral and lateral views (Fig. 5A, C), tapering from base to apex, phallicata tubular, curved slightly upwards in lateral view (Fig. 5C); about same length as phallobase (Fig. 5A, C).

##### Etymology.

The Latin feminine adjective *fasciaria*, meaning “banded,” refers to the band of white hairs across each wing in dorsal view.

##### Distribution.

China (Hainan).

#### ﻿ Paduniella sanyaensis

Taxon classificationAnimaliaTrichopteraPsychomyiidae

Peng & Sun sp. nov.

10567567-EBBF-53FB-9E3D-7C651C622EED

https://zoobank.org/291BCBF2-64D5-4144-9B56-1533FE24E023

Fig. 6A–C

##### Type materials.

***Holotype***: China • 1♂; Hainan Province, Sanya City, Tianya district, Hongxin village, Hongxinxi River; 18°27.78'N, 109°27.88'E; alt. 150 m; 22-vii-2022; light trap; L. Peng & H. Zang leg.; NJAU Tricho-20220722-0001. ***Paratypes***: China • 2♂; same data as holotype; NJAU Tricho-20220722-0002 to Tricho-20220722-0003 • 2♂; Hainan Province, Sanya City, Tianya district, Fuwan reservoir; 18°16.80'N, 109°28.94'E; alt. 60 m; 25-vii-2022; light trap; L. Peng & H. Zang leg.; NJAU Tricho-20220725-0001 to Tricho-20220725-0002.

##### Diagnosis.

This species is similar to *Paduniellanama* Johanson & Oláh, 2010 from Vietnam. However, *P.sanyaensis* sp. nov. can be easily distinguished by the following characteristics: (1) the tergum IX is similar in width to the sclerotized strips in lateral view, whereas in *P.nama* tergum IX significantly wider than sclerotized strips; (2) the superior appendages are elongate-triangular in lateral view, but subrectangular in *P.nama*; and (3) the phallobase is enlarged, nearly triangular in lateral view, a feature missing in *P.nama*.

##### Description.

**Figure 6.**
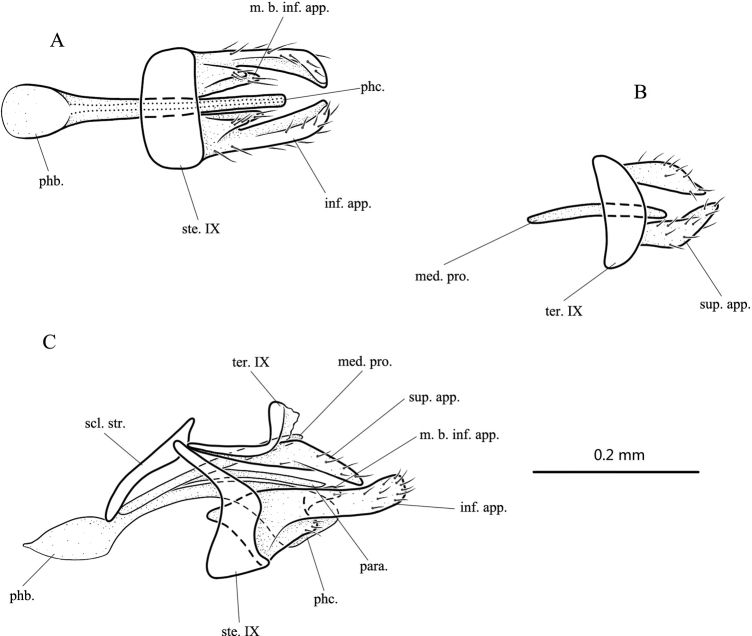
*Paduniellasanyaensis* sp. nov., male adult, holotype **A** genitalia, ventral **B** genitalia, dorsal **C** genitalia, left lateral. Abbreviations: ste. IX = sternum IX; ter. IX = tergum IX; sup. app. = superior appendage (paired); med. pro. = median process; inf. app. = inferior appendage (paired); m. b. inf. app. = mesal branch of inferior appendage (paired); para. = paramere; scl. str. = sclerotized strip; phb. = phallobase; phc. = phallicata.

**Male.** Length of each forewing 2.1–2.3 mm (*n* = 5), holotype 2.3 mm. Specimens in alcohol uniformly pale yellow-brown, antennae annulate with brown. ***Genitalia*.** Sternum IX in lateral view with lower portion subrectangular and upper portion produced anterodorsad on each side into slender lobe (Fig. 6C); in ventral view transversely subrectangular (Fig. 6A). Sclerotized portion of tergum IX L-shaped, upturned distally, distal end membranous in lateral view (Fig. 6C); somewhat crescentic in dorsal view (Fig. 6B). Superior appendages elongate-triangular in lateral view (Fig. 6C); lightly twisted and triangular in dorsal view (Fig. 6B). Sclerotized strips slightly clavate in lateral view (Fig. 6C). Median process slender, tubular, arising from anterior bases of sclerotized strips above phallobase (Fig. 6B, C). Inferior appendages each with basal 1/4 broad, then narrower, with upper and lower margins parallel to each other and slightly sinuous, apex truncate in lateral view (Fig. 6C); curved mesad with apex slightly enlarged in ventral view (Fig. 6A); mesal branch of each inferior appendage arising from middle part of inner surface, elongate-triangular in ventral view (Fig. 6A). Phallobase well developed, subtriangular, about half as long as phallicata in lateral view (Fig. 6C); subcircular in ventral view (Fig. 6A); phallicata curved posteroventrad, compressed, with apex enlarged in lateral view (Fig. 6C); slender in ventral view (Fig. 6A); paramere slender, shorter than phallicata in lateral view (Fig. 6C).

##### Etymology.

Latin feminine adjective *sanyaensis*, the name referring to the location of the type locality in Sanya City.

##### Distribution.

China (Hainan).

#### ﻿ Tinodes aviformis

Taxon classificationAnimaliaTrichopteraPsychomyiidae

Peng & Sun sp. nov.

035099CB-5738-5361-8E6F-044816353046

https://zoobank.org/6B523126-44D5-4152-BC2B-3C8971A42BCD

Fig. 7A–E

##### Type materials.

***Holotype***: China • 1♂; Hainan Province, Sanya City, Tianya district, Fuwan reservoir; 18°16.80'N, 109°28.94'E; alt. 60 m; 25-vii-2022; light trap; L. Peng & H. Zang leg.; NJAU Tricho-20220725-0003.

##### Diagnosis.

This species is similar to *Tinodesigok* Kimmins, 1955 from Malaysia in the composition and morphology of the male genitalia, but it can be distinguished by: (1) two unpaired inner branches of the phallic sheath processes, of which one is short and straight and the other one curved, whereas both branches are curved in *T.igok*; (2) the mesal digitate process of each coxopodite is shorter than the harpago, curved, and with a sharp apex in ventral view, but is undulated and almost the same length as the harpago in *T.igok*; and (3) the phallic guide is divided into two branches with the dorsal one extending backward beyond the coxopodites in lateral view, rather than having only one uncinate branch that is not longer than the coxopodites in *T.igok*.

##### Description.

**Figure 7.**
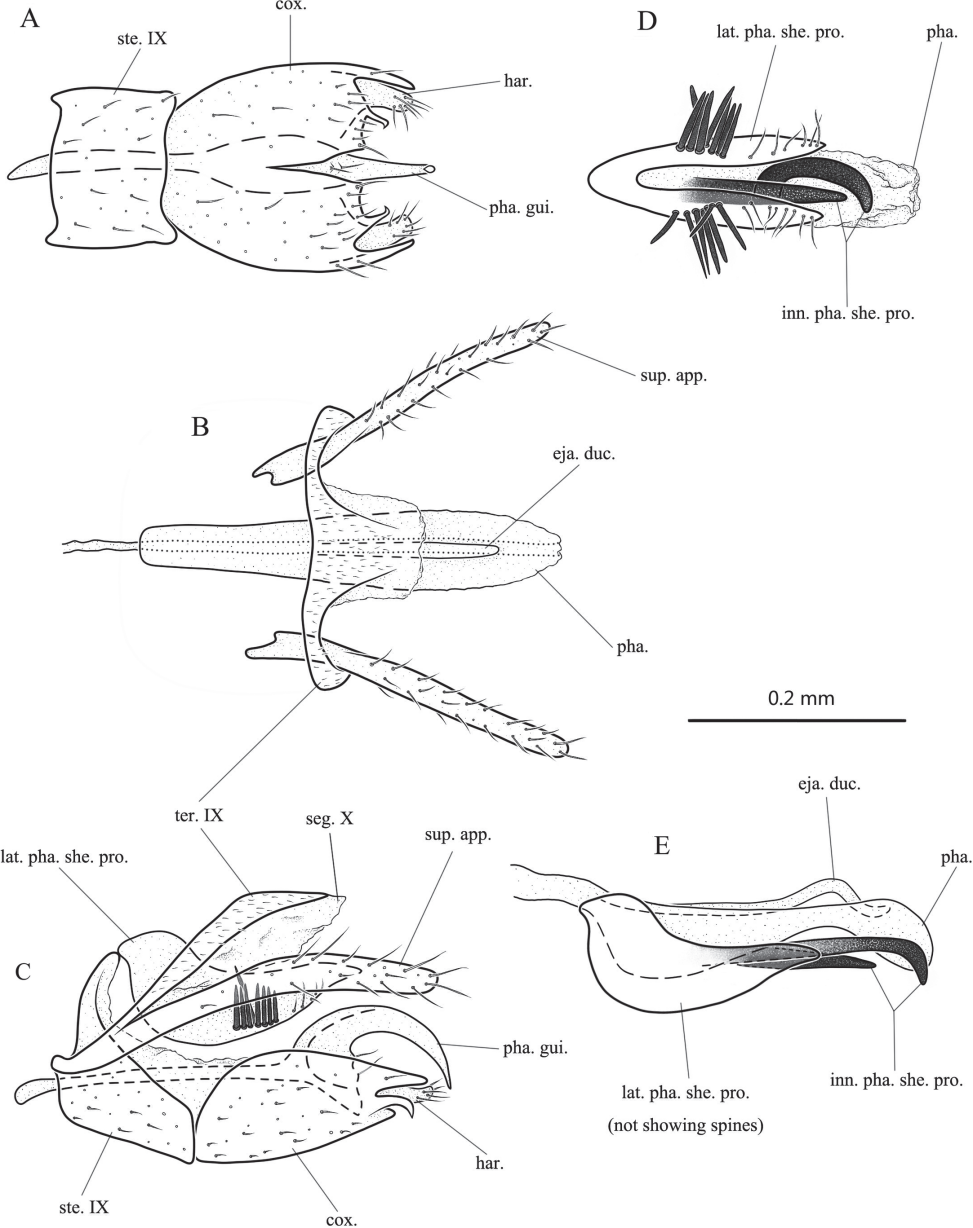
*Tinodesaviformis* sp. nov., male adult, holotype **A** genitalia, ventral **B** genitalia, dorsal **C** genitalia, left lateral **D** phallic complex, ventral **E** phallic complex, left lateral. Abbreviations: ste. IX = sternum IX; ter. IX = tergum IX; seg. X = segment X; sup. app. = superior appendage (paired); cox. = coxopodite (paired); har. = harpago (paired); pha. = phallus; pha. gui. = phallic guide; inn. pha. she. pro. = inner phallic sheath process; lat. pha. she. pro. = lateral phallic sheath process (paired); eja. duc. = ejaculatory duct.

**Male.** Length of each forewing 3.1 mm (*n* = 1). Specimen in alcohol with compound eyes black, antennae yellowish white; thorax and legs brown, wings light brown without any distinctive markings; abdomen dark brown dorsally, pale yellow ventrally. ***Genitalia*.** Sternum IX transversely subrectangular in ventral view (Fig. 7A); subtriangular in lateral view (Fig. 7C). Tergum IX covered with microchaetae, with anterior margin slightly sinuate and middle of posterior margin produced posterad in dorsal view (Fig. 7B); broader and subapically angled slightly caudad in lateral view (Fig. 7C). Segment X membranous, closely fused with tergum IX, its posterior margin nearly truncate in dorsal view (Fig. 7B); subrhomboid in lateral view (Fig. 7C). Superior appendages each with distal half setose, clavate, and with apex rounded in lateral view (Fig. 7C); parallel-sided in dorsal view (Fig. 7B). Phallic sheath process consisting of paired lateral branches and unpaired inner branches; paired lateral branches compressed and spoon-like, semicircular, and resembling nesting bird in lateral view (Fig. 7C, E), each with several strong spines at middle near ventral edge and distal half setose; fused basally, resembling pair of clam shells in ventral view (Fig. 7D); two unpaired inner branches strongly sclerotized (Fig. 7D, E); one of them straight, one curved; phallus slightly extending beyond tip of phallic sheath process, with distal end membranous in ventral view (Fig. 7D), base and apex swollen in lateral view (Fig. 7E); ejaculatory duct slender, with subapex S-shaped in lateral view (Fig. 7C). Phallic guide with subapex wider and then divided into two branches in lateral view (Fig. 7C), dorsal branch slender, curved downwards distally and gradually narrowed to sharp apex; ventral branch short. Coxopodites elliptical in lateral view (Fig. 7C); fused with each other basally in ventral view (Fig. 7A), each with posterodorsal angle digitate and posterior margin having tiny submesal digitate process; harpago setose, small, and simple (Fig. 7A, C).

##### Etymology.

The Latin masculine adjective *aviformis*, meaning “bird-shaped,” and refers to the shape of the pair of lobes of the lateral phallic sheath process in lateral view.

##### Distribution.

China (Hainan).

#### ﻿ Psychomyia indra

Taxon classificationAnimaliaTrichopteraPsychomyiidae

Malicky & Chantaramongkol, 1993

439C7EA8-F2E1-515B-BD86-5312067CC1FA

Psychomyia indra Malicky & Chantaramongkol, 1993: 1162 (type locality: Thailand, Tramot; ♂).

##### Material examined.

China – Hainan Province • 1♂; Ledong County, Jianfeng Town, Jianfengling National Forest Park, Rainforest Valley; 18°44.72'N, 108°56.08'E; alt. 640 m; 17-iv-2019; light trap; H. Song leg.; NJAU Tricho-20190417-0001.

##### Distribution.

China (Hainan [new record]); Thailand.

#### ﻿ Paduniella andamanensis

Taxon classificationAnimaliaTrichopteraPsychomyiidae

Malicky, 1979

FA352446-BDBE-5A57-981B-61FFFE0B3E29

Paduniella andamanensis Malicky, 1979: 98 (type locality: India, Süd-Andaman, Nayachul-Fluß bei Mongelutonge, Lichtfang [India, South Andaman, Nayachul River, near Manglutan, light trap]; ♂).

##### Material examined.

China – Hainan Province • 1♂, Yunnan Province, Xishuangbanna Dai Autonomous Prefecture, Jinghong City, Yunjinghong Street G214; 22°01.75'N, 100°52.12'E; alt. 660 m; 26-vii-2021; light trap; K. Jiang leg.; NJAU Tricho-20210726-0001.

##### Distribution.

China (Yunnan [new record]); India (Andaman Islands).

#### ﻿ Paduniella dendrobia

Taxon classificationAnimaliaTrichopteraPsychomyiidae

Malicky & Chantaramongkol, 1993

6A68AA44-EDC5-5EE1-B0AB-0C971A3E20D2

Paduniella dendrobia Malicky & Chantaramongkol, 1993: 1159 (type locality: Thailand, Doi Inthanon; ♂).

##### Material examined.

China – Sichuan Province • 1♂; Yibin City, Pingshan County, Xining River; 28°41.15'N, 103°45.97'E; alt. 370 m; 12-v-2020; X.Y. Ge & X. Chen leg.; NJAU Tricho-20200512-0001.

##### Distribution.

China (Sichuan [new record]); Thailand.

#### ﻿ Tinodes gapbona

Taxon classificationAnimaliaTrichopteraPsychomyiidae

Johanson & Oláh, 2008

43057C86-7B90-5973-BC6D-7D2F755D27D4

Tinodes gapbona Johanson & Oláh, 2008: 7 (type locality: Vietnam, Hoabinh towards Dabac; ♂).

##### Materials examined.

China – Guangdong Province • 2♂; Huizhou City, Yuguishan Nature Reserve; 22°25.80'N, 113°26.39'E; alt. 290 m; 17-ix–22-x-2020; Malaise trap; Institute of Zoology, Guangdong Academy of Sciences leg.; NJAU Tricho-20201022-0001 to Tricho-20201022-0002 • 20♂; Zhaoqing City, Dinghushan National Nature Reserve; 23°09.50'N, 112°32.46'E; alt. 170 m; 9-ix–9-x-2021; Malaise trap; Institute of Zoology, Guangdong Academy of Sciences leg.; NJAU Tricho-20201009-0001 to Tricho-20201009-0020.

##### Distribution.

China (Guangdong [new records]); Vietnam.

#### ﻿ Psychomyia imamiah

Taxon classificationAnimaliaTrichopteraPsychomyiidae

Malicky, 2020

DE7CA069-A596-5A20-95B5-AC0DEA8A27BC

Psychomyia polyacantha Li, Qiu & Morse, 2021 (in Qiu and Morse 2021) syn. nov. (type locality: China, Sichuan Province, Pingwu County, tributary of Fu-Jiang, 19 km E of Pingwu downtown, 32°24.72'N, 104°45.49'E, alt. 1090 m; ♂).

##### Materials examined.

China – Sichuan Province • 200+♂; Pingwu County, tributary of Fujiang, 19 km E of Pingwu downtown; 32°24.72'N, 104°45.49'E; alt. 1090 m; 27-vi-1990; J.C. Morse leg.; NJAU Tricho-19900627-0001 to Tricho-19900627-0200 • 200+♂; Pingwu downtown; 17 km E of Ping-wu trib. of Fujianghe; 32°24.48'N, 104°44.36'E; alt. 1090 m; 27-vi-1990; L. Yang & Y. Li leg.; NJAU Tricho-19900627-0201 to Tricho-19900627-0400 • 16♂, Kangding County, Guzazhen Town, Dadu River, Wasigou, at suspension footbridge, across the river from G318 at 2819.9 km stone marker 30°04.53'N, 102°09.61'E, alt. 1430 m, 29-vi-2005, Coll. X. Zhou, CJ Geraci leg.; NJAU Tricho-20050629-0001 to Tricho-20050629-0016 – Gansu Province • 6♂; Wenxian County, Bikou Town, Bifeng Gully; 32°44.72'N, 105°14.64'E; alt. 650 m; 16-vi-1998; L. Yang leg.; NJAU Tricho-19980616-0001 to Tricho-19980616-0006.

##### Distribution.

China (Sichuan, Gansu).

##### Remarks.

The specimens that we examined included topotypes that were collected at the same time as the type specimen of this species (Qiu and Morse 2021). We found that the harpagones each had four apical processes, which is rare in *Psychomyia*, occurring only in *P.polyacantha* and *P.imamiah*. By comparing these specimens and published descriptions and figures of the male genitalia of these two species, we believe that the morphological characteristics of the two species overlap. The type locality of *P.polyacantha* is in the basin of the Fujiang River, and the Fujiang River flows into the Jialing River, and the paratypes of *P.polyacantha* and the holotype of *P.imamiah* are from the basin of the Dadu River, which merges into the Minjiang River. Both the Jialing and Minjiang rivers are tributaries of the Yangtze, and their upstream habitats are similar and geographically adjacent. In summary, we identify *P.polyacantha* as a synonym of *P.imamiah*.

## ﻿Discussion

The Psychomyiidae is a moderately sized family of caddisflies (Holzenthal et al. 2007). However, the number of species has increased rapidly in recent years as new species are discovered and named; in 2008 there were about 400 species (de Moor and Ivanov 2008), in 2019 there were about 600 species (Morse et al. 2019), and now there are more than 650 species (including this study). Though the members of this family are widespread over nearly all the world (Holzenthal et al. 2007), the family’s species diversity is uneven across zoogeographical regions. Psychomyiids are found mostly in the Oriental and Palaearctic regions, with the combined total number of species in those two regions constituting more than 90% of the world’s species for this family. Of the two regions, the Oriental Region is the only one with all eight psychomyiid genera present, and it has about 65% of the world’s recorded species, significantly higher than the number of Palaearctic species, which constitute about 25% of the species among six genera (*Psychomyia*, *Tinodes*, *Paduniella*, *Metalype*, *Eoneureclipsis*, and *Lype*). Additionally, the Afrotropical region has three genera (*Tinodes*, *Paduniella*, and *Lype*) and 6.9% of the world’s species, the Nearctic region has four genera (*Psychomyia*, *Tinodes*, *Paduniella*, and *Lype*) and 2.8% of the world’s species, and the Australian region has only one genus (*Tinodes*) and 1.5% of the world’s species. China covers parts of the Oriental and Palaearctic regions and has more than 111 species in six genera (including those in this study), constituting about 17% of psychomyiid species, of which six genera (*Psychomyia*, *Tinodes*, *Paduniella*, *Metalype*, *Eoneureclipsis*, and *Lype*) and 100 species are recorded in China’s Oriental region, and four genera (*Psychomyia*, *Tinodes*, *Paduniella*, and *Metalype*) and 18 species are recorded in its Palaearctic region.

**Figure 8.**
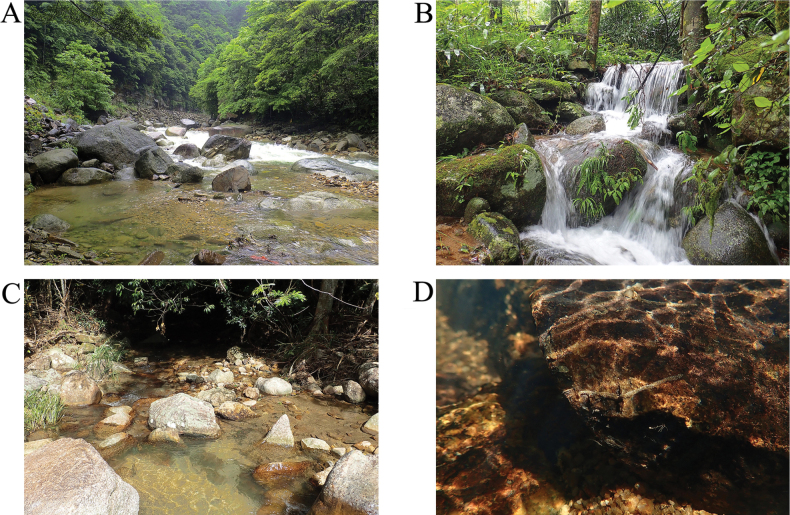
Photographs of habitat **A** a slow-flowing stream in the upper reaches of Yaorenping Hydropower Station, in Hunan Province **B** a rapids flowing stream in the upper reaches of Yaorenping Hydropower Station, in Hunan Province **C** a temporary pond in Jianfengling National Forest Park, Rainforest alley, in Hainan Province **D** the retreat of Psychomyiidae larvae and substratum composition of Hongxinxi River, in Hainan Province.

Most of the species in our study were collected from low latitudes, with a few from mid- latitudes (Table 1). Collection sites are near the source of streams or near a reservoir. Headwater streams had high forest cover and were small and cool, which is consistent with most psychomyiid larvae living in cool running water (Fig. 8A–D). While *Tinodesaviformis* sp. nov. was collected near a reservoir, similar to the habitats of some *Tinodes*, which have been reported to live in isolated stream pools in western North America and in lake littorals in Europe (Flint 1964; Wiggins 1996).

The male genitalia of the family vary among psychomyiid genera. In some genera, such as *Paduniella* and *Lype*, the structures of the male genitalia are simple enough to use common terms for describing them; however, in other genera, for example, in the genus *Tinodes*, extra structures are present in the genitalia, resulting in the different understanding on their homology, and accordingly, the terminology for the structures has varied among authors. The situation hinders phylogenetic study of the family based on morphology. We sincerely hope that, with phylogenetic studies using DNA sequences, the homology of these extra structures will become more generally understood and consensus for these terms will one day be reached, making interpretation of the evolution of the morphology and functional traits of these interesting and ecologically important animals more reliable.

## Supplementary Material

XML Treatment for Psychomyia shuni

XML Treatment for Psychomyia mangshanensis

XML Treatment for Psychomyia capricornis

XML Treatment for Lype sagittalis

XML Treatment for Paduniella fasciaria

XML Treatment for Paduniella sanyaensis

XML Treatment for Tinodes aviformis

XML Treatment for Psychomyia indra

XML Treatment for Paduniella andamanensis

XML Treatment for Paduniella dendrobia

XML Treatment for Tinodes gapbona

XML Treatment for Psychomyia imamiah

## ﻿Additional information

### Author ORCIDs

Lang Peng https://orcid.org/0000-0002-5644-8248

Zhen Deng https://orcid.org/0009-0004-4611-908X

Yu-hua Zhang https://orcid.org/0009-0001-8259-4038

Meng Wang https://orcid.org/0000-0001-5903-3171

Chang-hai Sun https://orcid.org/0000-0003-4061-1028

Bei-xin Wang https://orcid.org/0000-0002-5253-8799

### Data availability

All of the data that support the findings of this study are available in the main text.
